# Characterization of the Genomic Architecture and Mutational Spectrum of a Small Cell Prostate Carcinoma

**DOI:** 10.3390/genes5020366

**Published:** 2014-05-12

**Authors:** Alan F. Scott, David W. Mohr, Hua Ling, Robert B. Scharpf, Peng Zhang, Gregory S. Liptak

**Affiliations:** 1 McKusick-Nathans Institute of Genetic Medicine, Johns Hopkins University School of Medicine, Baltimore, MD 21287, USA; E-Mails: dwmohr@jhmi.edu (D.W.M.); hling1@jhmi.edu (H.L.); pzhang13@jhmi.edu (P.Z.); 2 Department of Oncology, Johns Hopkins University School of Medicine, Baltimore, MD 21287, USA; E-Mail: rscharpf@jhsph.edu; 3 Department of Pediatrics, SUNY Upstate Medical Center, Golisano Children’s Hospital, Syracuse, NY 13210, USA; E-Mail: gliptak@rochester.rr.com

**Keywords:** small cell prostate cancer (SCPC), exome sequencing, Illumina SNP array, Ingenuity, NGS (next generation sequencing)

## Abstract

We present the use of a series of laboratory, analytical and interpretation methods to investigate personalized cancer care for a case of small cell prostate carcinoma (SCPC), a rare and aggressive tumor with poor prognosis, for which the underlying genomic architecture and mutational spectrum has not been well characterized. We performed both SNP genotyping and exome sequencing of a Virchow node metastasis from a patient with SCPC. A variety of methods were used to analyze and interpret the tumor genome for copy number variation, loss of heterozygosity (LOH), somatic mosaicism and mutations in genes from known cancer pathways. The combination of genotyping and exome sequencing approaches provided more information than either technique alone. The results showed widespread evidence of copy number changes involving most chromosomes including the possible loss of both alleles of CDKN1B (p27/Kip1). LOH was observed for the regions encompassing the tumor suppressors TP53, RB1, and CHD1. Predicted damaging somatic mutations were observed in the retained TP53 and RB1 alleles. Mutations in other genes that may be functionally relevant were noted, especially the recently reported high confidence cancer drivers FOXA1 and CCAR1. The disruption of multiple cancer drivers underscores why SCPC may be such a difficult cancer to manage.

## 1. Introduction

The promise of the Human Genome Project (HGP), for which we mark the tenth anniversary, was that individualized genomics would become a reality for medical diagnosis and care. However, only recently have methods for sequencing, data analysis and the interpretation of variation with respect to medically relevant sequence information become sufficiently robust to make this approach useful. In this paper we compared different methods to investigate the genomic architecture and mutational spectrum of a rare tumor, small cell prostate cancer (SCPC). Our goal was to identify which methods were most informative and what information might provide the best guidance to the patient and his physician. Secondarily, we hoped to provide further characterization for this tumor type that may be of use to the community.

SCPC is a high-grade malignant tumor with neuroendrocrine differentiation sometimes referred to as neuroendocrine prostate cancer (NEPC) [1]. SCPC is often discovered after the occurrence of metastases, has been reported to account for 0.5%–2% of all prostate carcinomas and has a median survival from diagnosis of approximately 12.5 months [2]. The largest SCPC series was published by Wang and Epstein [3] who histologically examined 95 cases of which 92% showed expression of the neuroendocrine marker CD56 (NCAM1) and of which approximately 80% failed to show elevated PSA levels. Aparicio *et al.* [1] noted that, although rare as a primary diagnosis, NEPC may be more common than appreciated and could account for as much as 25% of lethal prostate cancer.

A few studies have looked at the genomic events characterizing NEPC. Beltran *et al.* [4] measured gene expression using NGS RNA-sequencing and oligonucleotide arrays in NEPC tumors and observed a correlation between overexpression of MYCN and AURKA both of which were amplified at the gene level in 40% of NEPCs. The authors also noted evidence for a TMPRSS2-ERG gene fusion, a lack of the ERG protein marker, high expression of the neuroendocrine genes CGA and SYP, and low expression of the androgen-regulated genes KLK3 (PSA), TMPRSS2 and NXK3.1. Beltran *et al.* [4] further showed that NEPC cell lines were sensitive to the AURKA inhibitor danusertib which produced a suppression of neuroendocrine expression. However, phase II clinical trials of men with castration-resistant prostate cancer were disappointing [5]. Tzelepi *et al.* [6] produced SCPC xenografts and performed expression studies and genomic profiling using array-CGH (comparative genomic hybridization) which showed up-regulation of UBE2C and other mitotic genes along with the absence of expression of the androgen receptor (AR), RB1, and cyclin D1. A subset of tumors showed microdeletions of RB1. Grasso *et al.* [7] sequenced 50 lethal metastatic castration-resistant prostate cancers (CRPC) which include SCPC. The authors identified subsets of tumors with either disruptions in CHD1 (chromodomain helicase DNA-binding protein 1) or in ETS2 (usually from fusions of ETS2 with TMPRSS2). The authors also found mutations in multiple genes whose protein products physically interact with androgen receptors such as the ERG gene fusion, the chromatin modifying protein MLL2, and FOXA1 among others. Grasso *et al.* [7] further showed that mutated FOXA1 repressed androgen signaling and enhanced tumor growth. The importance of FOXA1 in tumor progression was also demonstrated by Imamura *et al.* [8] who were able to reduce proliferation in cell culture with an siRNA directed against FOXA1. Van Allen *et al.* [9] performed whole exome sequencing on a CRPC bone metastasis and identified a homozygous deletion in PTEN and a nonsense mutation in BRCA2, both of which suggested clinical treatment strategies.

The diversity of findings in the studies cited above likely results from both tumor heterogeneity and the different laboratory methods used. In this study we have used array based genotyping to examine the overall genomic architecture, exome sequencing to identify somatic mutations, various software tools to analyze the resulting data and both public and commercial interpretation tools to attempt to understand the findings.

## 2. Experimental

### 2.1. Sample

The patient was a consented 63-year old male of European ancestry who presented with hematuria and without elevated levels of prostate specific antigen (PSA). The cancer was detected after the development of metastases and the diagnosis of SCPC was made at the patient’s primary care hospital and confirmed at the Johns Hopkins Hospital. High molecular weight DNA was isolated from two needle aspirates of a metastatic Virchow node and from saliva (Scope^TM^ mouthwash, Procter & Gamble) using standard methods but with extended proteinase K digestion time for the biopsies. Cells from the aspirates were examined by a pathologist at the time of collection and the remaining tissue was transferred, unfixed, to the laboratory for DNA isolation. A total of 25.5 µg of DNA was obtained from the tumor and 60 µg from the mouthwash collection.

### 2.2. Genomic SNP Array and Analysis

**Figure 1:**
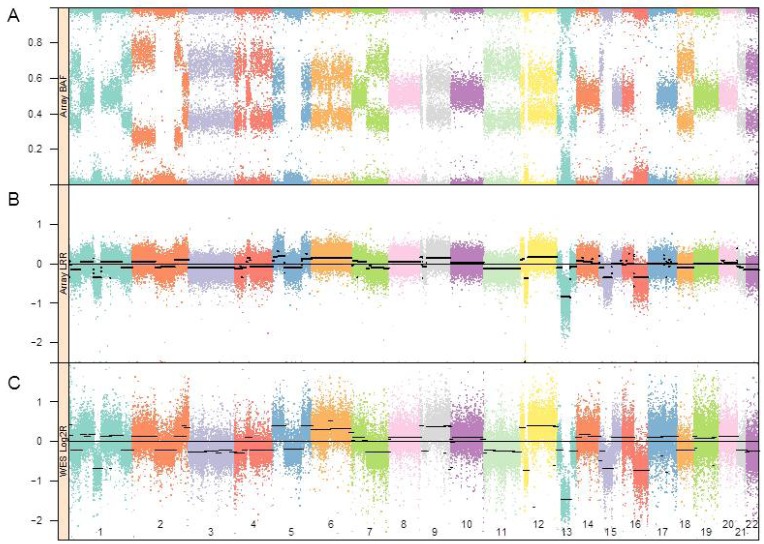
Allele frequencies (**A**) and log R ratios (**B**) estimated by GenomeStudio. Autosomal log R ratios were segmented by circular binary segmentation [11] as indicated in black; (**C**) Log R values from whole exome sequencing were obtained by the EXCAVATOR program [12] and aligned to panels A and B, providing a qualitatively similar profile of the copy number alterations. Black lines depict the segmentation of the log R values.

The DNA from the metastasis was adjusted to 50 ng/µL and 5 µL (250 ng) were genotyped on an Illumina HumanOmni2.5S BeadChip^TM^ array at the SNP Center of the Genetic Resources Core Facility [10] at the Johns Hopkins School of Medicine. Illumina GenomeStudio software (Illumina Inc., San Diego, CA, USA) was used to process the array data and calculate B-allele frequencies (BAF) and log R Ratios (LRR). The BAF and LRR values generated by GenomeStudio ver. 1.7.4 (Illumina, Inc.) are plotted in Figure 1 (panels A and B, respectively) for all the autosomes. The LRR values were segmented using the circular binary segmentation algorithm implemented in the R package DNAcopy (ver. 1.36.0) [11]. The black lines in the LRR plots are the average LRRs for those segments of the chromosome.

### 2.3. Exome Capture and Sequencing

Exome-sequencing was performed at the High-Throughput Sequencing facility of the GRCF. DNA (3 µg) from the tumor and saliva were sheared to a size of 150 to 200 bp using a Covaris E210 system (Covaris Inc., Woburn, MA, USA). End repair and addition of an overhanging “A” base was performed using a NEBNext^TM^ reagent kit (New England Biolabs, Ipswich, MA, USA). DNA fragments were ligated to library adapters (Illumina). The ligated fragments were then size selected through purification using SPRI beads and PCR amplified to prepare the libraries. An Agilent Bioanalyzer DNA1000 assay was used for quality control of the libraries to ensure adequate concentration and appropriate fragment size. Sequencing was performed on an Illumina HiSeq^TM^ 2000 following library capture with an Agilent SureSelect All Exon v3 kit. Sample indexing was applied to distinguish the source of the libraries. Sequence data was processed using CIDRSeqsuite v2.3.0 [13] as follows. Sequence reads were processed through Illumina software generating base calls and corresponding base-call quality scores. Resulting data was aligned to hg19 with the Burrows-Wheeler Alignment (BWA; [14]) tool resulting in a SAM/BAM file. Molecular and optical duplicate reads were flagged using software from the Picard program suite [15]. Post-processing of the aligned data included local realignment around SNPs and indels and base-call quality score recalibration using the Genome Analysis Tool Kit ver. 2 (GATK2; [16]). Single sample calling was done using GATK2 HaplotypeCaller with hard filtering and outputted in VCF 4.0 format. Analyses were performed in accordance with GATK Best Practices recommendations [17,18]. All positions reported are with respect to the hg19 reference sequence.

### 2.4. Sequence Interpretation

Differences of SNVs and indels between the tumor and normal exomes were computed using both open source and commercial software to identify somatic mutations. The open source programs included ANNOVAR [19], SG-adviser, a suite of web-based tool offered by The Scripps Translational Science Institute [20] Strelka [21] and Seurat [22] which were used with their default settings. In addition, we used the IntOGen-mutations platform [23,24] to identify genes mutated in the TCGA/ICCG cancer genome projects. The Condel online tool [25] was used to obtain scores for missense mutations [26] shown in the exome sequencing data tables. We also used the Ingenuity Variant Analysis web-based application to compare sequence between the tumor and matched normal exomes with differing filtering parameters (see Supplementary Methods: Analysis and Variant Filtering).

## 3. Results and Discussion

### 3.1. Genomic Landscape Detailed from Genotyping Data

**Figure 2:**
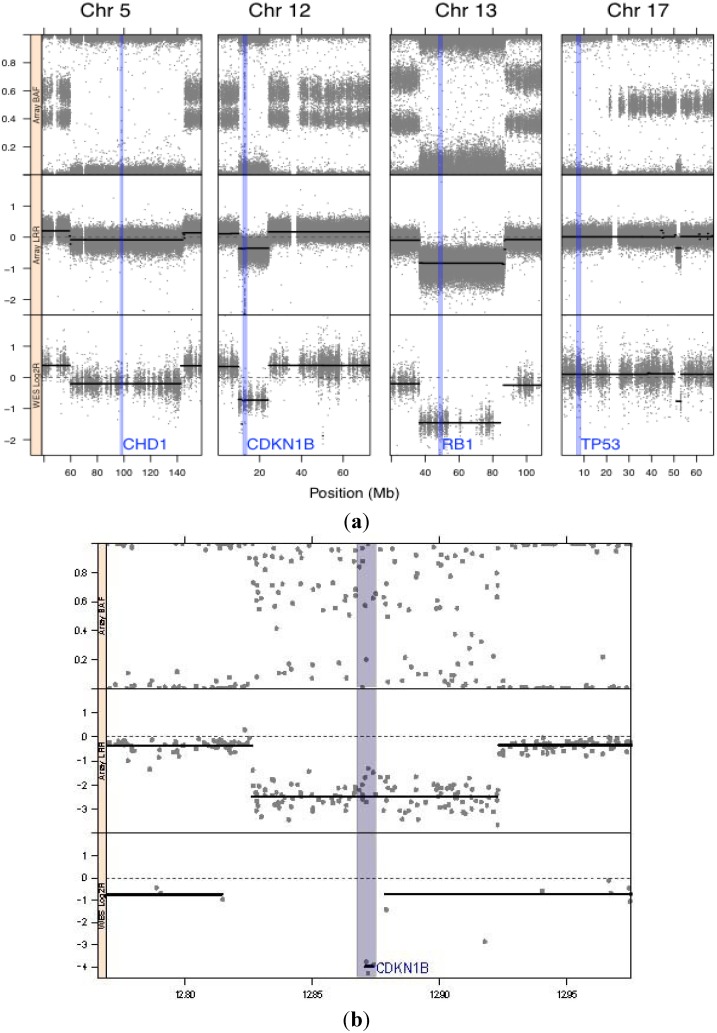
The location of the key genes described in the text. CHD1 (Chr 5: 98.190–98.265 Mb) was not mutated but occurs in a block of LOH; CDKN1B (Chr 12: 12.870–12.875 Mb) has low copy number, RB1 (Chr 13: 48.878 Mb–49.056 Mb) occurs in a block of LOH, has reduced copy number and the retained allele is predicted to be damaging; TP53 (Chr 17: 75.712 Mb–75.909 Mb) occurs in a copy neutral block of LOH and the retained allele is predicted to be damaged. Figure 2b, magnified view of the CDKN1B region showing a likely deletion of the gene supported by both the array (top and middle panels) and exome-sequencing platforms (bottom).

The genotyping array showed a large degree of copy number variation of chromosomal segments and loss of heterozygosity with 17 of the 22 autosomes grossly affected (Figure 1). The genotypes from the Illumina 2.5 M BeadChip processed by circular binary segmentation indicated a modest range in copy number. Visual inspection of the BAF plots clearly identifies blocks of homozygosity on chromosomes 1, 2, 5, 9, 12, 13, 15, 16, and 17. At least four critical tumor suppressors are within these regions of LOH; TP53 on Chr 17, RB1 on Chr 13, CDKN1B on Chr 12 and CHD1 on chromosome 5 (Figure 2a). While the regions that include TP53 and CHD1 are essentially copy neutral, the LRR plot and segmentation showed a reduced copy number for the chromosome 13 block containing RB1 and a likely homozygous deletion of the chromosome 12 region containing CDKN1B (Figure 2b).

Grasso *et al.* [7] recently described the mutational landscape of castration-resistant prostate cancer (CRPC) based on exome sequencing of 50 lethal metastatic cases. An important finding of their study was that tumors involving CHD1 lacked ETS2 gene fusions and ETS2 mutations. CHD1 is an ATP-dependent chromatin-remodeling enzyme that recognizes histone H3 lysine 4 methylation and is associated with the promoters of active genes where it presumably acts in nucleosome disassembly [27]. In this tumor the regions around ETS2 and TMPRSS2 have normal copy number although SNP arrays are not capable of identifying contiguous chromosomal events and a translocation in non-coding DNA is certainly possible. In contrast, CHD1 clearly falls within a region of LOH and although the exon sequences for the gene are the same as reference we do not know if there might have been mutations in regulatory regions. Unfortunately, we were unable to obtain RNA from the limited biopsy specimen so we could not measure changes in CHD1 expression.

The genotyping data identified a region of LOH and markedly reduced copy number on chromosome 12p (~Chr 12: 10–24 Mb) that includes CDKN1B (Chr 12: 12.870–12.875 Mb) the gene which encodes the p27 cyclin-dependent kinase inhibitor also referred to as p27(KIP1). The exome reads were also significantly reduced and it is possible that the gene is completely absent in the tumor and that the observed reads represent those from contaminating or infiltrating normal cells. CDKN1B blocks cell division in G_0_/G_1_, regulates cell motility and apoptosis and is classified as a tumor suppressor [28]. Although CDKN1B was not mutated in this tumor, decreased copy number has been associated with tumor pathology in mice (e.g., [29]) and in lethal human epithelial cancers with a poor outcome [30].

Tumor Purity

Several regions of LOH identified by genotyping showed very few spurious mutant sequencing reads indicating that normal cells were not present in the tumor to any significant degree (e.g., TP53 Chr 17:5,578,394). Nevertheless, the mean read depth for the tumor library was 353X and 138X for the normal exome. Although the range in the frequency of mutant reads (Table 1) varies considerably, the fact that 97% of the reads for TP53 are mutant confirms that the tumor was unlikely to have been contaminated with a significant number of normal cells and that differences in allelic fraction at other positions most likely represent tumor heterogeneity. Because of this high level of tumor purity we felt that our exome sequencing provided a good representation of the genomic events without the need to sequence to extraordinary depth to distinguish tumor from infiltrating non-tumor cells.

### 3.2. Exome Variant Interpretation

We used open source tools to generate variant calls and a mixture of open-source and commercial programs to evaluate the significance of the somatic mutations. All variant filtering is a trade-off between sensitivity and specificity and the risk of missing variants of biological significance must be weighed against a larger number of false calls. The variant calling programs included Haplotype Caller [16,17], Strelka [21] and Seurat [22]. Haplotype Caller was run under GATK best practices with hard filtering [18], producing single sample calls for both tumor and normal. Strelka and Seurat are somatic variant callers that identify SNVs and indels present in a tumor but not the matched normal sample. Both were run using default settings. The default filter for Seurat removes sequences with a mapping quality score less than 10 while Strelka removes all read pairs with a mapping quality below 40. Seurat identified 3577 somatic SNVs and 2290 indels. In comparison, Strelka found 535 SNVs and 11 indels. Lists from both somatic callers were submitted to the Integrative Onco Genomics single tumor analysis web tool [23] which searches somatic mutations, genes and pathways identified, at the time of the analysis, from 4623 tumor/normal exomes by the International Cancer Genome Consortium (ICCG; [31]) and The Cancer Genome Anatomy (TCGA; [32]) initiatives. Because the Seurat results appeared to have high sensitivity but low specificity we decided to focus on the Strelka list. We manually inspected the Strelka /IntOGen dataset by examining each of the positions using the Integrative Genomic Browser (IGV, ver. 2.3.23; [33]).

**Table 1:** Principal findings: Protein coding genes with somatic mutations. The copy number and location within regions of LOH are noted. The SNV in the tumor *vs.* normal cells is indicated in the allele column and the percent of variant reads or mosaicism is given. Condel scores (D = deleterious, N = neutral) and protein changes are shown for each predicted isoform. Premature stop mutations are indicated and assumed to be damaging. Genes confirmed by the Ingenuity analysis are indicated along with the Ingenuity “assessment”.

Chr	Position	Gene	Driver	CN	EXC	LOH	Ref	SNV	% Var	Condel Score	Protein change	Ingenuity Assesment	Gene Description	Comments
17	7,578,394	TP53	HCD	2.02	2.12	YES	C	T	0.97	D(0.97–1.0)	H179R, H86R, H47R	Pathogenic	Tumor protein p53	Damaging in all alternate translation products
13	48,941,657	RB1	HCD	1.12	0.72	YES	G	T	0.75	STOP GAIN	E323*	Pathogenic	Retinoblastoma 1	Premature termination codon is inferred damaging
14	38,061,334	FOXA1	HCD	2.11	2.25	NO	G	T	0.44	D(1.0)	R219S, R186S	Likely Pathogenic	Forkhead box A1	Damaging in two alternate translation products
10	70,508,917	CCAR1	HCD	2.05	2.13	NO	G	A	0.24	N(0.02), D(0.81), D(0.83), N(0.02)	R269H, R258H, R284H, R89H	No Assessment	Cell division cycle and apoptosis regulator 1	Probably damaging in two alternate translation products
12	12.870 Mb– 12.875 Mb	CDKN1B	HCD	0.36	0.13	YES		None		Same as hg19 reference	DELETION	Not flagged	Cyclin-dependent kinase inhibitor 1B (p27/KIP1)	No mutations in coding regions

Ingenuity Variant Analysis (QIAGEN, Redwood City, CA, USA) was also used to filter and interpret somatic variants under different filtering criteria and single-sample variant call files were uploaded, parsed, and comparatively queried initially for rare (<3% allele frequency in public genome/exome datasets) missense, nonsense, coding indel, or clinically classified (pathogenic/likely pathogenic) variants, confidently called (PHRED-scaled variant call quality >20 in either sample) in genes directly or indirectly (within 2 upstream interaction hops) implicated in “prostate cancer” or “small cell adenocarcinoma” (interactive supplement at https://variants.ingenuity.com/Scott-etal-2014). Other filtering parameters (e.g., 1 upstream interaction hop, frequency in 1000 genomes or Complete Genomics data of less than 0.001%, and broader disease terms including “small cell adenocarcinoma”, “castration-refractory prostate cancer”, and “metastases”) were also evaluated (interactive supplement at https://variants.ingenuity.com/Scott2014ver2).

We grouped SNVs into four categories: (1) The principal findings (Table 1) that we speculate have a strong likelihood of causing or contributing to SCPC; (2) Potentially implicated genes (Table 2) for which there is some evidence of an involvement in cancer but are less certain; (3) Genes with probable passenger mutations (Table S1) whose involvement in cancer is less obvious or lacking and (4) Possibly inherited risk factors (Table S2) for cancer susceptibility. The distinction between each of the first three categories is somewhat arbitrary.

Table 1 shows the top six genes based on their designation as high-confidence or candidate drivers (HCD, CD) of cancer by Tamborero *et al.* [34], their consensus deleteriousness (Condel) scores [26] or their reduced copy number. Premature nonsense mutations were presumed to be deleterious. The fraction of mutant reads at each position was also calculated from the BWA alignment. As discussed above, the predicted copy numbers in Table 1 and whether the gene fell into a region of LOH was based on both the genotyping array data as well as normalized exome capture read depth. 

The most obvious findings from the genotyping and sequencing data are that the classic tumor suppressors TP53 and RB1 both occur in blocks of LOH and the retained alleles were mutated. In the case of TP53 the His to Arg missense substitution is damaging by both SIFT and Condel. The RB1 mutation was a premature stop codon at amino acid 323. As with other treatment resistant cancers, mutations in TP53 and RB1 have been reported in lethal prostate cancers [6,7]. Mutations in MLL2 have been reported in about 9% of CRPC while mutations in FOXA1 occurred in about 3.4% of tumors [7]. In this case of SCPC we did not find mutations in MLL2 but did detect a mutation in FOXA1. FOXA1 is a nuclear protein that promotes tumor progression through its interaction with the androgen receptor, which in turn, induces several prostate-specific genes. FOXA1 levels are positively correlated with PSA, Gleason scores and AR expression [8]. The damaging FOXA1 mutation reported here occurred in about half of the sequence reads and is presumed to result in lower activity which may, in part, explain the fact that the patient’s PSA levels were not elevated. Grasso *et al.* [7] also showed that FOXA1 mutations repressed androgen signaling and increased tumor growth. 

**Table 2:** Potentially implicated genes. Selection criteria included whether the gene function is cancer-related, a member of a gene family with established cancer drivers, or assessed as damaged by Condel or the Ingenuity Variant Caller.

Chr	Position	Gene	CN	EXC	LOH	Ref	SNV	% Var	Condel Score	Protein change	Ingenuity Assesment	Gene Description	Comments
2	106,498,240	NCK2	1.89	1.70	YES	C	G	1.00	D(0.91)	P228R	Uncertain	NCK adaptor protein 2	Promotes melanoma cell proliferation, migration and invasion
2	107,041,278	RGPD3	1.89	1.70	YES	C	A	0.90	SIFT = Damaging	E1049*	Likely Pathogenic	RANBP2-like and GRIP domain containing 3	Reported expression in testis and HeLa cells
1	109,742,795–109,742,798	KIAA1324, EIG21	1.57	1.24	YES	G	4 bp del	0.85	Frameshift	G829fs*10	Uncertain	KIAA1324; Estrogen induced gene 121	High expression is associated with shorter survival in ovarian cancer
2	102,407,183	MAP4K4	1.89	1.70	YES	G	T	0.49	D(0.88) or N(0.02)	G42V, G4V	Likely Pathogenic	Mitogen-activated protein kinase kinase kinase kinase 4	Often overexpressed in cancer and has roles in various cancer processes
19	50,247,621	TSKS	2.00	2.14	NO	C	T	0.45	N(0.05)	E410K	Likely Pathogenic	Testis-specific kinase substrate	Low expression in some embryonal carcinoma lines
15	88,678,358	NTRK3	2.02	2.14	NO	C	T	0.41	D(0.72–0.87)	G295D, G393D	Likely Pathogenic	Neurotrophic tyrosine kinase, receptor, type 3	Potential tumor suppressor, often fused with ETV6 in thyroid cancer
16	7,759,062	RBFOX1	2.10	2.14	NO	G	A	0.40	D(0.91–1.0)	G307R, G334R, G355R, G339R, G377R	Uncertain	RNA binding protein, fox-1 homolog (*C. elegans*) 1	Related gene RBFOX2 is a Candidate Driver (CD)
19	38,865,389	PSMD8	2.00	2.10	NO	C	T	0.38	N(0.00)	R50C	Uncertain	Proteasome (prosome, macropain) 26S subunit, non-ATPase, 8	Upregulated in a choriocarcinoma cell line
12	31,254,871	DDX11	2.25	2.61	NO	C	G	0.34	N(0.37)	H693Q, H317Q	Likely Pathogenic	DEAD/H box helicase 11	Associated with small-cell carcinoma
1	36,290,920	AGO4	1.83	1.71	NO	G	A	0.24	N(0.00)	M105V	Uncertain	Argonaute RISC catalytic component 4	Down-regulated in hepatocellular cancer
12	31,250,875	DDX11	2.25	2.61	NO	G	C	0.14	D(0.49)	A607P	Pathogenic	DEAD/H box helicase 11	Expressed at high levels in melanoma
19	4,689,651	DPP9	2.00	2.09	NO	G	T	0.12	D(0.50)	S560R	Uncertain	Dipeptidyl-peptidase 9	Expressed in breast and ovarian cancers
10	81,921,760	ANXA11	2.05	2.13	NO	G	A	0.04	D(0.85–0.95)	R338C, R371C, R4C	Not flagged	Annexin A11	May enhance metastasis and invasion; related gene ANXA6 is a Candidate Driver (CD)
9	100,843,284	TRIM14	2.23	2.59	NO	C	T	0.03	D(0.82–0.94)	R264W	Not flagged	Tripartite motif containing 14	Related gene TRIM7 is a High Confidence Driver (HCD)

Barbieri *et al.* [35] examined 112 prostate cancer tumor-normal pairs by exome sequencing and found recurrent somatic mutations in the genes FOXA1 and MED12 (~5% of tumors each) and in SPOP (~13% of tumors) in individuals with metastatic disease. The authors observed three different FOXA1 missense mutations in the forkhead (FH) domain, the DNA-binding domain of the protein [35,36]. FOXA1 binds to the androgen receptor and regulates the transcription of prostatic genes and is required for development of the prostate. The damaging mutation reported here also occurred in the FH domain. We did not observe mutations in SPOP and it does not occur in the region of LOH we observed on chromosome 17. Likewise, MED12 on the X chromosome did not appear to have somatic mutations when compared to the normal DNA sample.

Recurrent deletions of 5q21 have been reported in prostate cancer [35,37], and correlated with loss of the tumor suppressor CHD1. Further, Burkhardt *et al.* [37] showed a strong correlation between the loss of CHD1 and the biochemical failure to detect prostate-specific antigen. Similarly, we observed a region of LOH on chromosome 5 (~60–145 Mb, 5q12.1–31.3) which includes the CHD1 gene (Chr 5: 98,188,908–98,264,238) and, as noted, PSA levels were also not elevated in this cancer. While the remaining allele of CHD1 appears to have a normal sequence the genotyping array shows a reduced LRR. We were unable to perform studies to determine if RNA or protein levels were concomitantly reduced. The LOH region in our patient also includes the PIK3R1 gene, mutations in which are associated with various tumors (e.g., [9,38]). We observed no somatic mutations in the retained PIK3R1 allele.

The list of potentially implicated genes with somatic mutations is shown in Table 2 and ordered by the percent of mosaicism of the variant. These were selected based on the Ingenuity assessment, being a member of a gene family in which a related gene is a known or candidate cancer driver or from published literature implicating them in some aspect of cancer. Among these are NCK2 whose potentially damaging mutation occurs in 100% of reads. NCK2 is reported to promote melanoma cell proliferation, migration and invasion [39]. RGPD3 is expressed in the testis and HeLa cells [40], KIAA1324 or estrogen-expressed gene 21 is associated with ovarian cancer survival [41] and overexpression of EIG121 was observed to cause “profound suppression” of cell growth [42]. Presumably, reduced expression would have the opposite effect. MAP4K4 is a serine/threonine kinase that is overexpressed in many cancers where it is implicated in migration and invasion [43] and RBFOX1 is related to the candidate driver RBFOX2 [44]. Zhou *et al.* [45] reported a mutation in RBFOX1 in a colorectal adenoma. Decreased expression of testis-specific kinase substrate, TSKS has been observed in cancerous testicular tissue and in very low levels in various embryonal carcinomas [46]. NTRK3 is a potential tumor suppressor [47] often fused with ETV6 in thyroid cancer [48]. PSMD8 is up-regulated in a choriocarcinoma cell line [49]. AGO4 or EIF2C4 is down-regulated in hepatocellular cancer [50]. DDX11 is required for sister chromatid cohesion and is expressed at high levels in primary and metastatic melanomas [51] and DDP9 is expressed in breast and ovarian cancer [52]. ANXA11 plays an important role in cell division and disruption of the gene “may lead to or enhance the metastasis, invasion and drug resistance of cancers” [53]. A literature survey for TRIM14 did not identify a link to cancer but the protein shares homology to the reported high confidence driver TRIM7 [34].

We noted LOH and slightly reduced copy number for the region on chromosome 2 (181.5–181.8 Mb) containing the long non-coding RNAs SChLAP1 (LINC00913) [54] and for PCGEM1 (193.6 Mb) [55] both of which have been reported to be overexpressed in aggressive prostate cancer. We did not find evidence that CDKN2A was deleted or that CCNE1, E2F3, UBE2C, or MYCC were amplified as seen in other cancers (e.g., [56]). Our patient did not receive castration therapy and the androgen receptor gene (AR) was not deleted. We did not find evidence for a fusion between TRPSSC2 and ERG based on exon sequences although, as noted above, we cannot rule out a translocation outside of coding regions. Cyclin D1 (CCND1), a gene often altered in cancer and a modifier of androgen receptor function [57], may have reduced copy numbers based on the array data but had no obvious sequence differences from the normal sample. We also found no evidence for copy number or somatic mutations in AURKA, KLK3, CGA, SYP, NXK3.1, NCAM1, CD56, ETS or UBE2C. In fact, the total estimated mutational burden is low (<1/50,000 bp).

The patient’s normal genome was also studied for inherited SNPs that might confer an increased risk for cancer (Table S2). A heterozygous SNP in FOXC1 that creates a P321Q variant that is predicted to be deleterious by SIFT and Condel (score = 0.92) was observed. Overexpression of FOXC1 has been correlated with poor outcome (e.g., [58,59]) and as a promoter of invasion in breast cancer [60]. The patient was also heterozygous for a known rare variant in DND1 (rs72800920) that is predicted to be damaging by both SIFT and CONDEL (score = 0.96). In mice, a premature stop mutation in Dnd1 has been shown to markedly increase the risk of testicular germ line tumors [61]. We do not know if rs72800920 is a cancer risk factor or simply a private rare variant. Other possible risk factors for which the patient was heterozygous were ALK, NCK2, DDX11 and CBWD3. Somatic mutations in ALK (anaplastic lymphoma receptor tyrosine kinase) have been seen in neuoblastomas [62]. NCK adaptor protein 2, NCK2, has been reported to promote melanoma cell proliferation [39]. DEAD/DEAH box helicase 11, DDX11, is required for sister chromatid cohesion and has been reported to be essential for the survival of advanced melanomas [51]. It is curious that the patient was a carrier for a likely pathogenic inherited variant and his tumor showed two somatic mutations in DDX11. Each of the germline alleles in these genes remained heterozygous in the tumor (*i.e.*, did not show evidence for selection) and we have no formal evidence that they conferred risk for disease or its progression.

## 4. Conclusions

The main goal of this study was to assess how the new genomic technologies, analysis methods and interpretation tools might be used to provide clinical utility. Secondarily, we hoped to better characterize a SCPC metastasis in a single case using these approaches. The combination of genotyping arrays, to provide a broad overview of the genomic landscape, and exome sequencing, to identify specific mutations, was more useful than either method alone. The genotyping array highlighted key regions of the genome that showed abnormal copy number or loss of heterozygosity. In general, these changes in genomic architecture are clues to underlying genes that may be implicated in cancer. LOH is commonly associated with the loss of tumor suppressors and by identifying those regions first on an array we were able to focus attention on the somatic sequence variants found there.

Because of limited sample we were unable to perform karyotyping or expression studies (either arrays or RNAseq). However, such limitations are likely to be expected in routine clinical testing so maximizing information from samples is critical. Going forward it would be preferable to do dual RNA and DNA isolations from fresh needle biopsies and perform RNA sequencing to measure relative expression levels, identify the main splice variants and any fusion transcripts. Given the good correlation between exome read depth and copy number from the array shown here it may be unnecessary to perform high-density genotyping in the future. However, we would likely replace exome-capture with PCR-free whole genome sequencing in order to eliminate biases related to the capture reagents and be able to potentially identify chromosomal translocation events and other genomic rearrangements. Currently, we feel it is important to manually review the sequence data at key positions and perform Sanger sequencing as confirmation for actionable mutations. However, improvements in laboratory methods and analysis may soon make this unnecessary (e.g., [63]).

We found that both open source and commercial tools were invaluable for interpreting somatic variants although it is essential that the analysis pipeline that produces the variants for interpretation be as rigorous as possible. Interpretation software essentially performs two tasks: it matches lists of variants within a study to those reported in various databases or in the literature in a way that is meaningful for the disease or mode of inheritance and it uses one or more algorithms to predict the effects of mutations. The first function will only be as good as the databases referenced and for commercial databases the details are usually not available. In general, there was excellent concordance between the open source tools and the Ingenuity Variant Analysis although the latter identified several somatic mutations in genes that were not flagged by the IntOGen analysis. This is not surprising given that IntOGen is based on data from large cancer sequencing projects while Ingenuity also includes literature-based gene information and predictive algorithms that infer change in protein function.

A current limitation to variant interpretation is, as seen in Table 1, that many genes produce multiple alternative transcripts and may have deleterious mutations predicted in some isoforms but not others. In the absence of RNASeq data we do not know which isoforms may predominate in a given cancer type. Information about the splice variants and fusion transcripts will have to be included in a comprehensive analysis. Further, because the interpretation tools used in this analysis were based on VCFs they did not take copy number or LOH into account. As shown from the Excavator analysis this is something that could certainly be added. A clear advantage of the Ingenuity Variant Analysis tool was the ability of a user to easily link to the biomedical literature and pathway information that included potential drugs for targets it identified. Providing such information will be a valuable adjunct to physicians acting on exome and genome test results.

As noted above, as with other types of genetic testing, NGS approaches need to be standardized, accurate and have practical utility. Perhaps more than other genetic tests, whole genome or whole exome sequencing blurs the borders between clinical testing and research. This was also true of other methods when they first appeared (e.g., FISH, comparative hybridization arrays, *etc.*) and only with the accumulation of large datasets and more standardized methods in the laboratory and during analysis will the utility of genome sequencing become routine. As more correlations are made between patterns of the genomic landscape and mutational profiles we should be better able to tailor treatments or predict the course of disease. Already, sequencing data are being used to design patient-specific tests to follow response to treatment [64] and gene or mutation-specific treatments have been and are being developed.

SCPC is a lethal cancer with a poor prognosis. Ciriello *et al.* [56], in summarizing the ICGC and TCGA oncogenic signatures from over 3000 tumors, concluded that cancers generally fall into one of two classifications; “M” class cancers with, often large numbers of somatic mutations, and the “C” class with chromosomal abnormalities and fewer variants but which often involve somatic mutations in TP53, the likely cause of the genomic instability [65]. This tumor clearly falls into the C class. It is remarkable that while the overall somatic mutation rate was relatively low given that so many cancer driver genes were mutated. Perhaps the rarity of SCPC reflects the need to accumulate many separate deleterious mutations. Unfortunately, nothing in our sequencing or interpretation analysis offered a useful treatment strategy but, hopefully, the approaches and results reported here will be of use to others studying this and other aggressive cancers. By redefining cancers based on their genomic, expression and mutational architectures we may be able to markedly improve cancer diagnosis and therapy.

## Supplementary Files

Supplementary File 1 Supplementary Information (PDF, 164 KB)
