# Supplementary material for: Characterization of the Genomic Architecture and Mutational Spectrum of a Small Cell Prostate Carcinoma
Source: Genes (Basel). 2014 May 12;5(2):366–84. doi: 10.3390/genes5020366 (PMC4094938; doi:10.3390/genes5020366)
Supplement: Supplementary File 1 — Supplementary Information (PDF, 164 KB) [file genes-05-00366-s001.pdf]

Supplementary Information

Table S1. Likely passenger genes with somatic mutations ranked according to the percent of variant reads per site. Condel scores are indicated as deleterious (D) or neutral (N) with the computed score.

Chr	Position	Gene	CN	EXC	LOH	Ref	SNV	% Var	Condel Score	Protein Change	Ingenuity Assessment	Gene Description
3	11,300,889	HRH1	1.87	1.68	NO	C	T	0.60	D(0.99)	R56W	Likely Pathogenic	Histamine Receptor H1
16	87,723,471	JPH3	1.58	1.21	YES	C	A	0.88	STOP GAIN	S502*	Uncertain	Junctophilin 3
12	129,180,383	TMEM132C	2.25	2.58	NO	G	A	0.58	D(0.51)	R171H, R555H	Not flagged	Transmembrane protein 132C
19	40,730,438	CNTD2	2.00	2.10	NO	C	A	0.51	D(0.95–0.99)	C127F, C157F, C51F	Not flagged	Cyclin N-terminal domain containing 2
1	62,190,653	TM2D1	2.08	2.23	NO	T	G	0.50	N(0.01)	K47T	Uncertain	TM2 domain containing 1
19	20,807,802	ZNF626	2.00	2.10	NO	A	C	0.48	D(0.89-1.0)	F294C	Likely Pathogenic	Zinc finger protein 626
4	52,861,657	LRRC66	2.07	2.15	NO	T	C	0.46	N(0)	R511G	Uncertain	Leucine rich repeat containing 66
19	14,710,851	CLEC17A	1.85	2.00	NO	C	T	0.44	STOP GAIN	R251*, R234*	Not flagged	C-type lectin domain family 17, member A
17	42,288,264-86	UBTF	2.01	2.17	NO		22 bp del	0.32	Frameshift	S412fs	Uncertain	Upstream binding transcription factor, RNA polymerase I
11	124,266,700	OR8B3	1.85	1.65	NO	G	A	0.28	D(0.97)	P183L	Not flagged	Olfactory receptor, family 8, subfamily B member 8
22	29,445,317	ZNRF3	1.82	1.69	NO	C	T	0.28	D(0.84)	T383M, T283M	Uncertain	Zinc and ring finger 3
20	36,948,603	BPI	2.03	2.17	NO	C	A	0.26	N(0.22)	S232Y	Uncertain	Bactericidal/permeability-increasing protein
19	58,797,099	ZNF8	2.00	2.01	NO	G	A	0.25	D(0.81)	R28Q	Likely Pathogenic	Zinc finger protein 8
15	41,820,531	RPAP1	1.57	1.23	YES	C	A	0.23	D(0.5)	A388S	Not flagged	RNA polymerase II associated protein 1
3	148,904,325	CP	1.87	1.71	NO	T	G	0.23	D(0.91), N(0.05)	M687L, M470L	Likely Pathogenic	Ceruloplasmin (ferroxidase)
19	49,227,680	RASIP1	2.00	2.14	NO	C	A	0.19	D(0.97)	G820W	Not flagged	Ras interacting protein 1
6	71,238,129	FAM135A	2.23	2.86	NO	T	C	0.16	D(0.89–0.99)	L1037P, L1054P, L1250P, L830P	Likely Pathogenic	Family with sequence similarity 135, member A
12	307,151	SLC6A12	2.16	2.53	NO	T	C	0.15	D(0.62–0.82)	T289A	Not flagged	Solute carrier family 6 (neurotransmitter transporter/GABA), member 12

Table S1. *Cont.*

Chr	Position	Gene	CN	EXC	LOH	Ref	SNV	% Var	Condel Score	Protein Change	Ingenuity Assessment	Gene Description
2	112,813,281	TMEM87B	1.89	1.70	NO	C	A	0.13	D(0.79)	A38E	Not flagged	Transmembrane protein 87B
7	100,243,891	ACTL6B	1.86	1.65	NO	C	T	0.05	D(1.0)	G394D	Not flagged	Actin-like 6B
20	43,739,136	WFDC5	2.03	2.17	NO	G	T	0.04	D(0.5–0.84)	P91H	Not flagged	WAP four-disulfide core domain 5
10	81,058,910	ZMIZ1	2.05	2.13	NO	C	A	0.04	D(0.83–0.85)	F590L	Not flagged	Zinc finger, MIZ-type containing 1
17	76,433,731	DNH17	2.02	2.18	NO	A	T	0.04	D(0.85), D(0.95), N(0.05)	F4003I, F4004I, F1210I	Not flagged	Dynein, axonemal, heavy chain 17
18	28,986,048	DSG4	1.87	1.71	NO	G	A	0.03	D(0.81)	A549T	Not flagged	Desmoglein 4
1	52,861,802	ORC1	2.08	2.18	NO	C	A	0.03	STOP GAIN	E213*	Not flagged	Origin recognition complex, subunit 1
6	147,636,696	STXBP5	2.23	2.48	NO	A	T	0.02	D(0.82–0.90)	E154V, E483V	Not flagged	Syntaxin binding protein 5 (tomosyn)

Table S2. Possible inherited cancer-related risk factors ranked according to Condel D score. All positions were heterozygous.

Chr	Position	Gene	Ref	SNV	Condel Score	Protein Change	Ingenuity Assessment	Gene Description	Comments
5	140,052,407	DND1	G	A	D(0.96)	P76L	Likely Pathogenic	DND microRNA-mediated repression inhibitor 1	Increases spontaneous testicular germ cell tumors in mice
2	29,416,181	ALK	A	G	D(0.94), N(0.01)	L421S, L1591S	Likely Pathogenic	Anaplastic lymphoma receptor tyrosine kinase	Likely pathogenic, oncogenic mutations seen in neuroblastomas
6	1,611,642	FOXC1	C	A	D(0.92)	P321Q	Uncertain	Forkhead box C1	High expression is associated with poor cancer prognosis
2	106,498,240	NCK2	C	G	D(0.91)	P228R	No assessment	NCK adaptor protein 2	Promotes melanoma progression
12	31,254,871	DDX11	C	G	D(0.64), N(0.37)	H693Q, H719Q	Likely Pathogenic	DEAD/H box helicase 11	Required for sister chromatid cohesion; seen in advanced melanoma
9	70,863,777	CBWD3	G	T	N(0.00)	D132Y	Likely Pathogenic	CBW domain containing 6	Mutations observed in COSMIC database (cancer.sanger.ac.uk)

Supplementary Methods: Analysis and Variant Filtering

Figure S1. Exome analysis pipeline used. Primary analysis was done using CIDRSeqSuite and tools from GATK. The final BAM files were submitted to Haplotype Caller for subsequent filtering or to either of two somatic variant callers (Seurat or Strelka). Interpretation of the results were determined from the Ingenuity Variant Analysis tool or from IntOGen web tool.

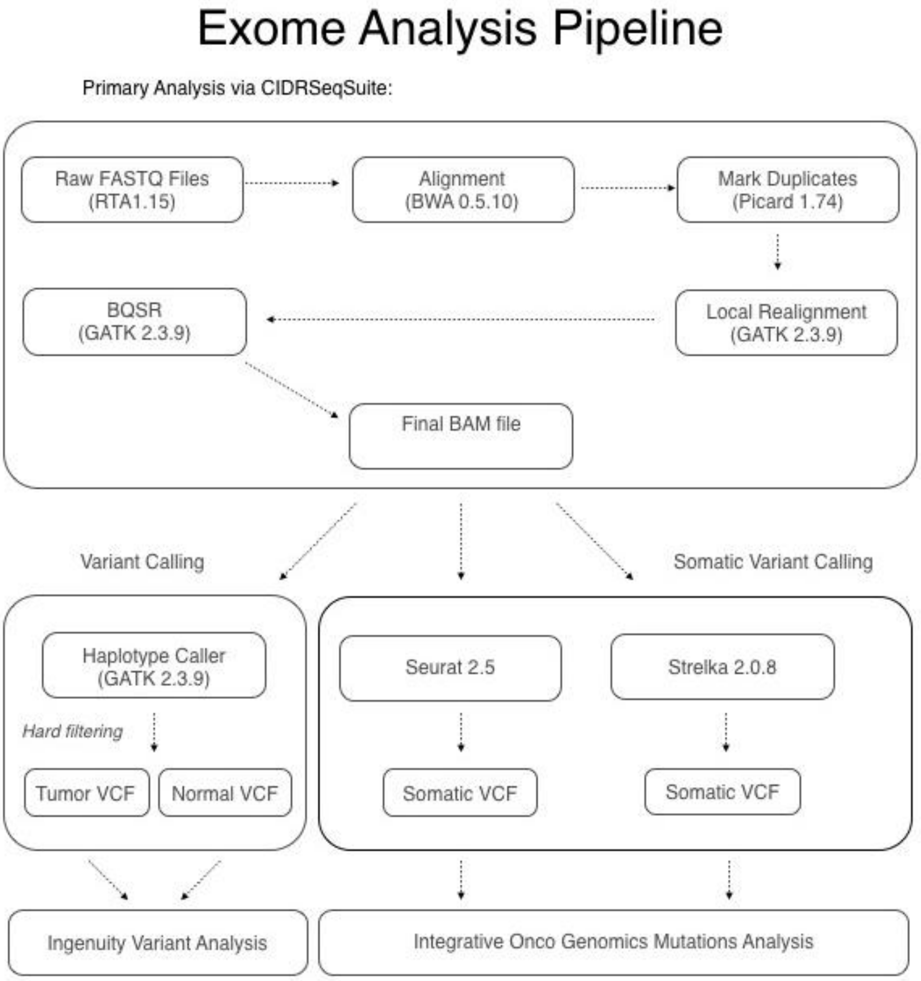

Variant detection was performed using GATK2.3.9 best practices to produce BAM files. Hard filtering of Haplotype Caller settings were as follows:

SNVs: QD < 2.0, FS > 60.0, MQ < 4.0, HaplotypeScore > 13.0, MappingQualotyRankSum < −12.5, ReadPosSum < −8.0; for Indels: QD < 2.0, FS > 200.0, ReadPosRankSum < −20.0.

Seurat 2.5 was run under default parameters.

Strelka 2.0.8 parameters:

isSkipDepthFilters = 1

maxInputDepth = 10000

depthFilterMultiple = 3.0

snvMaxFilteredBasecallFrac = 0.4

snvMaxSpanningDeletionFrac = 0.75
indelMaxRefRepeat = 8
indelMaxWindowFilteredBasecallFrac = 0.3
indelMaxIntHpolLength = 14 ssnvPrior = 0.000001
sindelPrior = 0.000001
ssnvNoise = 0.0000005
sindelNoise = 0.000001
ssnvNoiseStrandBiasFrac = 0.5
minTier1Mapq = 20
minTier2Mapq = 5
ssnvQuality_LowerBound = 15
sindelQuality_LowerBound = 30
isWriteRealignedBam = 0
binSize = 25000000

Ingenuity Variant Analysis parameters (http://variants.ingenuity.com/Scott-etal-2014):

The Ingenuity filtering was selected for increased sensitivity as opposed to maximum specificity. All SNVs listed in the Tables were visually inspected within IGV.

Filter Description:

Starting with 48248 variants spanning 13331 genes, variants were:

Kept with call quality at least 20.0 in cases or at least 20.0 in controls; excluded that are observed with an allele frequency greater than or equal to 3.0% of the genomes in the 1000 genomes project OR greater than or equal to 3.0% of the public Complete Genomics genomes OR greater than or equal to 3.0% of the NHLBI ESP exomes (All) kept that are experimentally observed to be associated with a phenotype: Pathogenic, Possibly Pathogenic OR Frameshift, in-frame indel, or stop codon change OR Missense.

Kept which are associated with gain of function OR heterozygous OR hemizygous OR homozygous OR nullizygous OR compound_heterozygous OR haploinsufficient AND occur in at least 1 of the case samples at the variant level in the Case samples AND not which are associated with gain of function OR hemizygous OR homozygous OR compound_heterozygous OR haploinsufficient AND occur in at least 1 of the control samples at the variant level in the Control Samples in at least 1 of the matched samples at variant level kept that are found in cancer-associated mouse knockout phenotypes OR cancer-associated cellular processes with appropriate directionality OR cancer-associated pathways with appropriate directionality OR cancer therapeutic targets OR published cancer literature variant and gene level findings OR TCGA at a frequency greater than or equal to 0.01% AND involved in: any cancer kept that are within 2 hops upstream and that are known or predicted to affect: prostate cancer or diseases consistent with these phenotypes.

Ingenuity Variant Analysis version 2.4.20140114.

Content versions: Ingenuity Knowledge Base (Zosma_131203.000), COSMIC (v67), dbSNP (Build 138 (08/09/2013)), 1000 Genome Frequency (v3), TargetScan (v6.2), EVS (ESP6500 0.0.21), JASPAR (10/12/2009), PhyloP hg18 (11/2009), PhyloP hg19 (01/2009), Vista Enhancer hg18. (10/27/2007), Vista Enhancer hg19 (12/26/2010), CGI Genomes (11/2011), SIFT (01/2013), BSIFT (01/2013), TCGA (09/05/2013), PolyPhen-2 (HumVar Training set 2011_12), Clinvar (10/01/2013).

Ingenuity Variant Analysis parameters (stricter filtering; https://variants.ingenuity.com/Scott2014ver2):

Filter Description:

Starting with 48248 variants spanning 13331 genes, variants were:

Kept with call quality at least 1.0 in cases or at least 1.0 in controls; excluded that are observed with an allele frequency greater than or equal to 0.0010% of the genomes in the 1000 genomes project OR greater than or equal to 0.0010% of the public Complete Genomics genomes OR greater than or equal to 0.0010% of the NHLBI ESP exomes (All).

Kept that are experimentally observed to be associated with a phenotype: Pathogenic, Possibly Pathogenic, Unknown Significance OR established gain of function in the literature OR gene fusions OR inferred activating mutations by Ingenuity OR predicted gain of function by BSIFT OR in a microRNA binding site OR in copy number gain genes OR Frameshift, in-frame indel, or stop codon change OR Missense OR disrupt splice site upto 2.0 bases into intron OR deleterious to a microRNA OR structural variant OR in promoter binding site OR in enhancer OR in evolutionary-conserved region with a phyloP *p*-value of less than or equal to 1.0E-6.

Kept which are associated with gain of function OR hemizygous OR haploinsufficient OR heterozygous OR heterozygous_alt OR heterozygous_amb OR compound_heterozygous OR nullizygous OR homozygous AND occur in at least 1 of the case samples at the variant level in the Case samples AND not which are associated with gain of function OR hemizygous OR haploinsufficient OR heterozygous OR heterozygous_alt OR heterozygous_amb OR compound_heterozygous OR homozygous AND occur in at least 1 of the control samples at the variant level in the Control Samples in atleast 1 of the matched samples at variant level.

Kept that are found in cancer-associated mouse knockout phenotypes OR cancer-associated cellular processes with appropriate directionality OR cancer-associated pathways with appropriate directionality OR cancer therapeutic targets OR published cancer literature variant and gene level findings OR known or predicted cancer subnetwork regulatory sites OR COSMIC at a frequency greater than or equal to 0.01% OR TCGA at a frequency greater than or equal to 0.01% AND involved in: any cancer.

Kept that are within 1 hop upstream and that are known or predicted to affect: small cell adenocarcinoma, castration-refractory prostate cancer (castration refractory prostate cancer process), prostate cancer, metastases or diseases consistent with these phenotypes.

Ingenuity Variant Analysis version 2.4.20140207.

Content versions: Ingenuity Knowledge Base (Zosma_131203.000), COSMIC (v67), dbSNP (Build 138 (08/09/2013)), 1000 Genome Frequency (v3), TargetScan (v6.2), EVS (ESP6500 0.0.21), JASPAR (10/12/2009), PhyloP hg18 (11/2009), PhyloP hg19 (01/2009), Vista Enhancer hg18 (10/27/2007), Vista Enhancer hg19 (12/26/2010), CGI Genomes (11/2011), SIFT (01/2013), BSIFT (01/2013), TCGA (09/05/2013), PolyPhen-2 (HumVar Training set 2011_12), Clinvar (10/01/2013).

© 2014 by the authors; licensee MDPI, Basel, Switzerland. This article is an open access article distributed under the terms and conditions of the Creative Commons Attribution license (http://creativecommons.org/licenses/by/3.0/).
